# Collaborative extended home-visits as a key to facilitating early support within the frame of a family centre in Sweden

**DOI:** 10.1186/s12913-024-12039-z

**Published:** 2024-12-03

**Authors:** Marie Golsäter, Ann-Christine Andersson

**Affiliations:** 1 CHILD Research Group, School of Health and Welfare, Jönköping University, Jönköping, Sweden; 2 The Child Health Care Service, Region Jönköping County, Jönköping, Sweden; 3 Futurum – Academy for Health and Care, Region Jönköping County, Jönköping, Sweden; 4 Jönköping Academy for Improvement of Health and Welfare, School of Health and Welfare, Jönköping University, Jönköping, Sweden; 5 Department of Care Science, Malmö University, Malmö, Sweden

**Keywords:** Child Health Services, Sunnybrook framework, Supportive structures, Team collaboration, Thematic analysis, Professionals’ experiences

## Abstract

**Background:**

All children should have the possibility to be healthy during childhood, according to the Convention on the Rights of the Child. In Sweden, the Child Health Services (CHS) support all parents and children from birth until the age of six to promote children’s health and development. Some Swedish regions have introduced an extended home-visit programme, with CHS nurses and social workers visiting first-time parents together to provide parental support in collaboration. The programme aims to expand the task of promoting the child’s health and increase the possibilities of discovering risk factors in families earlier. The aim of the present study is to describe the professionals’ experiences of collaboration when introducing the extended home-visit programme to a broader population within the frame of a family centre.

**Methods:**

The study used a reflexive thematic qualitative approach with focus group interviews. All staff at the family centre were invited to participate: CHS nurses, social workers, and managers who worked with the extended home-visit programme. Data were collected through focus group interviews with each profession separately and analysed through reflexive thematic analysis.

**Results:**

One overarching theme emerged: A key to facilitating early support. Three connected themes – Ease for everyone on the family’s terms, From working alone to becoming a team, and A matter of supporting structures – illuminated the participants’ experiences. Their driving force was early detection of risk factors or needs in the family, to be able to provide support. The collaboration was enhanced by the different professional competencies complementing each other. That all were located at the family centre together was also important to facilitate collaboration.

**Conclusions:**

The extended home-visits were appreciated and experienced as useful by all participants. That a family centre organization already existed was one of the facilitators, functioning as a meeting point to expand the collaboration. The managers’ support was essential, and it was experienced as positive that the organization invested resources to allow employees to participate in the development of the extended home-visit programmes. This way of working has the potential to add value for the children and families, and the CHS would benefit from using the extended home-visit programme further.

**Supplementary Information:**

The online version contains supplementary material available at 10.1186/s12913-024-12039-z.

## Background

The first years of a child’s life are essential to promote a healthy life [1, 2]. According to the Convention on the Rights of the Child [3], all children are entitled to the conditions for good health during childhood, and best possible health and development [1]. In Sweden, the Child Health Services (CHS) aim to support parents and promote children’s health and development, including all children from birth to the age of six [4]. The CHS’s services are voluntary and free of charge, and almost all parents choose to be included.

Based on proportional universalism [5], the national programme is built as a three-tier programme with universal interventions (tier I) and targeted interventions (tiers II–III) to offer all children and families good and equal care. Home-visits are part of the regular child health services, aiming to establish a caring relationship between the CHS nurse and the family [6]. The national programme comprises two universal home-visits by a CHS nurse, with the first when the child is newborn and the second at eight months of age. The CHS nurse can conduct more home-visits alone or with a social worker as a targeted intervention based on the family’s needs following the three-tier programme.

### Extended home-visits in Child Health Services

In recent years, some Swedish regions have started to conduct extended home-visits in collaboration by CHS nurses and social workers for first-time parents, based on the results from the Rinkeby programme [7], mainly in more socioeconomically vulnerable areas (see e.g. [8–11]). The extended home-visits aim to give families more universal visits and thereby expand the task of promoting the child’s health and increase the possibilities of discovering risk factors earlier. The intervention is founded on the World Health Organization’s (WHO) model for preventing unequal health due to socioeconomic disadvantage [12]. Further, the extended home-visit programme aligns with the WHO initiative on nurturing care to promote a safe childhood for every kid. Nurturing care is based on caregiving, protecting, preventing, and supporting children early in health, nutrition, safety, early learning, and responsible caring [13].

Research shows that extended home-visit programmes conducted by CHS nurses and social workers in collaboration also contribute to parents’ ability to build trust and trustful alliance towards Swedish authorities like the CHS and social services [14]. Further, parents describe how their homes, as a safe place, facilitate their receiving parental support more helpful and strengthen their parenting role [15].

### Teams and collaboration

Leaning on a theoretical micro system foundation [16], teams and collaboration are essential. Most CHS nurses and social workers are used to working independently with the families; therefore, introducing the extended home-visit programme entailed new working methods, including teamwork and close collaboration. Teams imply a group of people working together to accomplish a common task [17]; however, simply putting different professions in a group is not, by default, an interprofessional team. The results of the team’s performance must add value that each profession could not achieve on its own. The combined competencies should highly exceed the competence of a single profession, reaching collaborative gains [18].

Working in teams implies collaboration and is essential to improve care services and patient safety [19, 20]. Functional team collaboration can also improve patient and system outcomes [17]. A literature review shows that a well-designed team intervention enhances teamwork and improves outcomes [21].

Collaboration involves different actions, like communication, trust, respect, and understanding of each other’s roles and competences [22], aiming to maximize each party’s contribution to the common goal [23]. Social constructions, like different organizational belonging, mandates, and hierarchies, play a crucial role [23]. They can interfere with and counteract collaboration and prevent well-functioning teamwork, since teamwork requires an explicit will to collaborate and activities to be coordinated [18].

When establishing new teams, the Sunnybrook framework of core competencies for interprofessional team collaboration can be helpful [17, 24]. The framework consists of six core competencies for interprofessional team collaboration: 1) interprofessional values and ethics, 2) communication, 3) interprofessional conflict resolution, 4) reflection, 5) role clarification, and 6) shared decision making. Furthermore, interprofessional practice, interprofessional research and quality improvement, interprofessional leadership, and interprofessional training is included in the framework [17, 24].

### Local context

Region Jönköping County, located in southeast Sweden, is one of the 21 autonomous regions managing most of the healthcare for the country’s citizens. There are 13 municipalities in the region, almost 370,000 inhabitants, and about 26,000 preschool-aged children [25]. Each municipality has at least one family centre where midwives, CHS nurses, social workers, and preschool teachers work together. The region employs midwives and CHS nurses, while the municipalities employ social workers and preschool teachers. All CHS units in Region Jönköping County are organized as part of a family centre.

Working at a family centre involves teamwork and interprofessional collaboration [26]. Because all child health services in the region are organized as part of a family centre, there are promising opportunities for interprofessional teamwork. Within the scope of Region Jönköping County’s work Bästa platsen (Best Place To Live) [27] and building on the experiences from the Rinkeby programme [9, 28], in 2019 the CHS initiated the extended home-visit programme, adopted to each municipality’s resources, needs, and opportunities.

About a year after the extended home-visit programmes started, a pilot study was conducted as part of a Master’s thesis [29], consisting of focus group interviews with CHS nurses. The result showed that the CHS nurses experienced that the extended home-visits together with the social workers facilitated early support based on the families’ needs. The novelty with this extended home-visit programme is that it is offered to all families, not only those in a socioeconomic vulnerable area, which is common [7, 8, 14]. The present study wanted to further investigate the CHS nurses’ experiences as well as the social workers’ and managers’ experiences of working with the extended home-visit programme in frame of a family centre, covering all families within one municipality.

### Aim

The aim is to describe the professionals’ experiences of the introduction of an extended collaborative home-visit programme to a broader population within the frame of a family centre.

## Methods

### Setting

The municipality in focus here has about 31,899 inhabitants and an area of about 990 square kilometres. When planning for the extended home-visit programme, the available personnel resources and the structure of the family centre were considered. Additional personnel in the form of child health care nurses and social workers were added. In all, three social workers and nine child health care nurses worked at the family centre.

In collaboration between the managers from the CHS and social services, the CHS nurses, and the social workers, the extended home-visit programme was created that included four home-visits to all first-time parents and parents having their first child born in the municipality. All four home-visits were carried out together by a CHS nurse and a social worker, in contrast to the national CHS programme that offers two home-visits by a CHS nurse. The extended home-visit programme aimed to reach parents early in parenthood to offer relevant parenting support in an easily accessible way. The home-visits had different focuses, covering the issues *receive a child*, *togetherness in daily life*, *the role as a guiding parent*, and *to be a family*. The extended home-visit programme in Rinkeby offered six home-visits to all first-time parents in a socially disadvantaged area: when the child is newborn, two months, four months, eight months, twelve months, and fifteen months old [28].

The decision to offer four home-visits instead of six, as in the Rinkeby programme [28], was based on the available personnel resources. The extended home-visit programme was offered to all first-time parents in the municipality rather than just to families in a more socioeconomically vulnerable area (as in Rinkeby) because in this municipality, socioeconomic vulnerability was more scattered. To prevent families from feeling singled out or stigmatized [30], the managers, CHS nurses, and social workers jointly decided to offer the extended home-visit programme to all first-time parents and parents having their first child born in the municipality when the child is newborn, four months, eight months, and fifteen months old.

Based on the Rinkeby programme [9, 28], the CHS nurses, social workers, and managers started to adapt the project’s manual to the available resources and structures at the family centre. There was a lunch-to-lunch workshop with all staff and managers to plan for the design of the extended home-visit programme. As described in the Rinkeby manual, pictorial support used as call maps were constructed, and dolls were bought to be able to for example show child safety more concretely, or how to breastfeed, and how to carry a newborn. Further, the different schedules were adapted to customize times according to working hours and car access. Further, the CHS nurses and social workers were divided into micro teams comprised of one social worker and three CHS nurses each, with scheduled team coaching. Following the introduction of the extended home-visit programme, regular follow-ups were performed as half-day reflections, lunch-to-lunch workshops with the project leader and the managers, and follow-up meetings at the family centre. From the start on 1 September 2020 until 30 September 2022, about 560 extended home-visits were conducted as part of the programme.

### Design

The study uses a reflexive thematic qualitative approach [31] with focus group interviews [32].

### Participants

The study used a total population sample. All staff at the family centre were invited to participate. The participants were CHS nurses (*n* = 9), social workers (*n* = 3), and managers (*n* = 3) who had been working with the extended home-visit programme in the municipality. The CHS nurses were either specialists in paediatric nursing or in primary health care nursing. The social workers were educated in social science.

### Data collection

Data was collected through four focus group interviews with CHS nurses, social workers, and their managers. The interviews were conducted with each profession separately. Focus group interviews were applied to catch different perspectives on the participants experiences [32]. The Focus group interviews were carried out in the different professions separately due to the possibility to reflect on the collaboration between them. An interview guide was developed and used (supplemented file 1). Two groups of CHS nurses were interviewed in person (*n* = 3 and *n* = 5); one CHS nurse was on sick leave on the day of the focus group interview. Digital focus groups (using ZOOM®) were conducted with social workers (*n* = 3) and managers (*n* = 3). The focus group interviews were held in October 2022 by the second author. All interviews were recorded, lasted between 50 and 90 minutes, and were transcribed verbatim.

### Data analysis

Data was analysed using a reflexive thematic analysis [31]. This method offers a deliberative and reflexive approach when generating patterns of shared meaning [33]. The authors conducted the analysis both individually and together, reaching a consensus. To become familiar with the data, all transcripts were read several times. Data segments were identified and coded. The codes were then clustered into common areas, which indicated initial themes. The two authors discussed and revised the initial themes, and new themes arose. Through the revision process, themes were reflected upon and revied back against codes and the whole dataset. The core understanding of the data emerged through reflexive elaboration between the two authors on several occasions, and labels were generated to reflect the content. The themes were thoughtful and reflexive elaborated on, from a semantic to a more latent understanding, striving to refine the interpretations in a reflexive discussion [31]. A creative interpretation of the data was reached through the active work with codes and themes, and an overall concept was formulated: *a key to facilitating early support*. Quotations, used to strengthen the trustworthiness of the study, have been translated from Swedish by the authors and checked by a qualified language reviewer.

### Ethics

The study was performed in accordance with the Declaration of Helsinki and Swedish research regulations. The Swedish Ethical Review Authority approved the study, reference number 2021–04925. All participants consented in written to participate in focus group interviews.

## Results

**Fig. 1:**
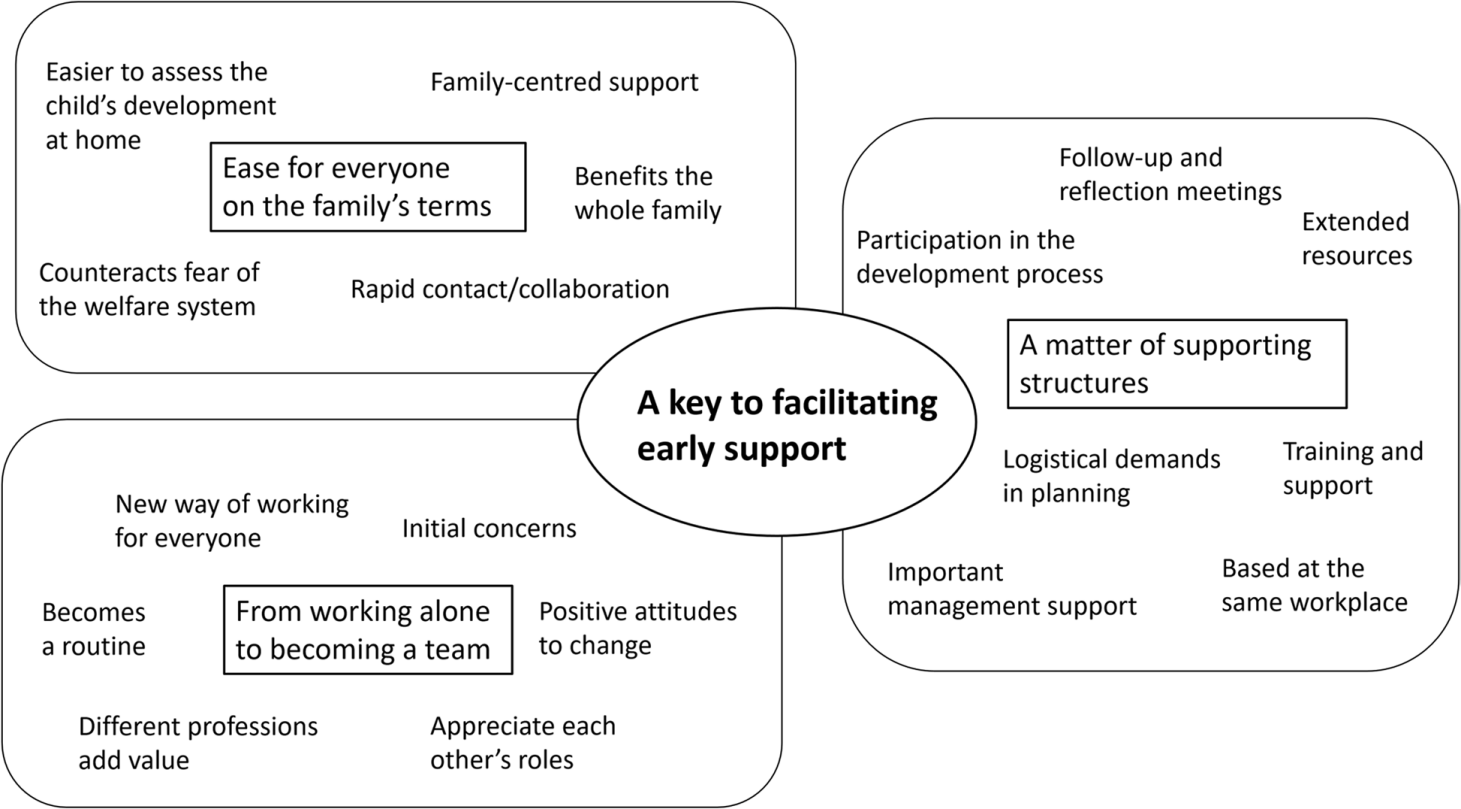
Result overarching theme, themes and their content

From the qualitative analysis, one overarching theme emerged: A key to facilitating early support. This includes three connected themes: Ease for everyone on the family’s terms; From working alone to becoming a team; and A matter of supporting structures (Fig. 1). The themes and the content are further developed below, illustrated with quotations.

### A key to facilitating early support

The participants clearly stated that the extended home-visit programme was a key to detecting needs and supporting families earlier if needed. The driving force was to improve, and if this could help detect any issues or problems earlier, it was appreciated by the CHS nurses, social workers, and managers. The CHS nurses were used to making home-visits independently, and social workers previously only came along if there were problems identified by the CHS nurse earlier. Offering a joint home-visit to all families was experienced as useful, since the extended home-visits provided a bridge to reach all families and provided opportunities to encounter their needs. The fact that the social worker was present from the beginning strengthened the possibilities of extended home-visits in creating a caring relationship as a starting point for the family’s continued contact with the family centre. The participants expressed some initial concerns that two different professions from different organizations coming home to the families could be experienced as frightening by the parents, but those concerns passed quickly after some home-visits. The benefits for all children and families getting better support than before, weighed up any issues. Working at the same place, the family centre, was also seen as a facilitating precondition. Before, if the CHS nurse wanted to discuss with a social worker, it was perceived to be much more complicated.

*“… if we need to bring in a social worker, yes, that step is so much easier now” (CHS nurse)*

The interpretations of the content in the three themes are developed below.

### Ease for everyone on the family’s terms

The participants were initially concerned that the social worker could be perceived as a control function, representing the authorities. Prior to the extended home-visit programme, there was a discussion about this. The CHS nurses and social workers felt able to express that the purpose of the visits was to make first-time parents more secure, and none of them experienced any concern from the parents during the home-visits. On the contrary, the participants expressed that the programme counteracted fear of the social care welfare system.

*“I have had families who have said: Now that you have been to our home and we have gotten to know you, I feel safe talking to you about those things” (Social worker)*

The participants also described that the fact that the CHS nurses and social workers were working as a team and were placed at the same location at the family centre were facilitating factors. Therefore, it did not matter that one was employed by the municipality and the other by the region, their organizational belonging was not important.

The participants described that the extended home-visits benefited the whole family; often, the parents had arranged that they both were home when the visit occurred. The participants also thought that it was easier to pay attention to both parents’ needs when they were two professionals. Being able to divide the work was experienced as a benefit of the teamwork. Even if one of them focused more on one thing, other issues were not forgotten, since the other one could focus on them. The CHS nurse paid more attention to the child and the breastfeeding mother, while the social worker had the opportunity to talk more deeply with the partner. This was experienced as making it easier for the partner to talk about things that bothered them, which otherwise were perceived as not to be talked about as a new parent. The parents were also perceived to be feeling safer in their own home, which made it easier for them to talk about their situation.

Another perceived advantage, expressed by the participants, was that if the parents needed additional support, the social worker could arrange that directly during the visit. Previously, the CHS nurses had to contact the social worker if the family needed extended support. Contacting the social worker was also easier now because they already knew each other.

*“The collaboration between the social care services and CHS has probably become even stronger, understanding each other’s activities and what we can achieve together in a really good and preventive way. This benefits the families and children” (Manager)*

Working in a team and visiting the family together was expressed by the participants as a potential facilitating factor for families to better understand that different professions at the family centre are working together to provide child health care and parental support.

The participants, both the CHS nurses and the social workers, appreciated the extended home-visits. The CHS nurses considered it easier to assess the child’s development in the home environment, familiar to the child, especially as the child gets older. The participants also highlighted that it is easier to talk about a safe environment for the child when at home.

*“It is much more relaxed when you go home to the families, where they are in their own [environment] and we come as guests. … Erm, and you get to know them, yes, you get to know them in a completely different way.” (CHS nurse)*

The CHS nurses and social workers thought they complemented each other when bringing up topics. In addition, the managers expressed that the staff seemed to be satisfied with the extended home-visit programme, and that it helped them work even more family-centred.

### From working alone to becoming a team

The participants thought that working in micro teams made the collaboration at the family centre easier also when other families, who were not included in the extended home-visit programme, expressed a need for extended support.

The extended home-visit programme was new to the CHS nurses and the social workers. There was a positive attitude to change, although some participants expressed that they were a little worried at first.

*“There has always been a desire for us to work closer together” (Manager)*

The participants expressed that the CHS nurses were used to doing home-visits on their own, but now, they were supposed to bring someone “watching” them. At the same time, the extended home-visits were aimed to be shared between three equal parties: the CHS nurse, the social worker, and the family. The CHS nurses feeling of “my” visit or “my” family was discussed in the preparation phase, introducing the work. Both CHS nurses and social workers needed to feel comfortable, which was expressed as being essential to support families in the best way. The participants described that they needed to reorganize the structure of the visits, as they had somewhat different content to convey.

*“… not been that easy, and collaborating is a challenge in itself” (Manager)*

The social workers expressed that it was vital that they were doing this as a team, not as them being “allowed to come” with the CHS nurses.

*“There were more concerns then, about what it would be like when we follow them [the CHS nurses]” (Social worker)*

The initial concern quickly changed when the participants experienced the advantages of working as a team and having someone to discuss and reflect with after the home-visit. The initial concerns about having someone watching you was replaced with a comfortable feeling of security; two persons notice more, which could be discussed in the car on the way back to the family centre.

*“Bringing someone with you for the first time who will be listening to what you say. Erm, so it was a bit nerve-wracking in the beginning … now I think it is fun to have the social worker with me. Now it almost feels weird when you need to go alone to a visit” (CHS nurse)*

In the beginning, all micro teams had team meetings together with a manager. The meetings allowed the teams to form, discuss their roles, and enhance collaboration. However, one team had stopped using such meetings, as they did not think there was a need anymore. This team had not changed any member. The teams that had changed personnel thought that the team meetings were crucial for forming the new team.

All participants expressed positive attitudes towards change in general, and particularly towards this change in the way of working. They all emphasized the importance of appreciating the different knowledge and roles they had, working together with someone as a team benefited the work, both on a professional level and for the family. Sometimes, the CHS nurses had to go on their own due to illness and difficulties to reschedule the appointment. In such cases, they missed having the opportunity to discuss things with the social worker and could not offer the family the social worker’s perspective. The participants perceived that being two different professions added competence and, therefore, added value for the family and to their team collaboration.

### A matter of supporting structures

The extended home-visit programme was introduced during a period that included informational meetings, getting to know one another, gatherings, training, and support from a project leader. This training and support were considered necessary for the positive attitudes from all participants. The managers emphasized the importance of having a project leader who could offer support in a way that they could not. The scheduled time and structure, such as reflective team meetings and follow-ups, were appreciated. This allowed for constantly improving the teamwork and the extended home-visit programme.

*“Here, they have dared to invest in this, and it’s because there have been driven managers who have been bold.” (CHS nurse)*

The participants thought that the planning and preparation phases before starting were essential for feeling confident about this new assignment. Further, all affected staff were involved early in the development and during the process. The process was not completed from the start but was designed in collaboration step by step. Another success factor was that there already existed a model to start from. Although adjustments were made, the basic elements in the model were followed.

The managers had to deal with the logistics, which was not always easy. CHC nurses and social workers also thought that the logistics was the hardest part to solve. If the social worker got sick, it was at times not possible to reschedule the visit, so sometimes the CHS nurses went by themselves. On the other hand, if the CHS nurse was sick, the home-visit might be cancelled and changed to a visit at the family centre.

*“… logistical problems are the main challenge” (CHS nurse)*

The teams differed in the extent to which they followed the planned schedule due to competing tasks, such as scheduled parenting groups and counselling. The fact that they were based at the same location at the Family centre was perceived as an advantage, it was easy just to talk with each other directly. They thought it would have been trickier if they had not worked at the family centre together.

*“That we are located at the same place, the family centre. It would not have worked if we had been located [at different places], at a health centre and a social welfare office. It would not have been the same at all” (Manager)*

During this period, the COVID-19 pandemic was a constraint, with restrictions and high levels of sick leave. However, the participants described how they prioritized the extended home-visit programme and were able to carry out the home-visits with support from the Department for Communicable Disease Control in the region.

When the project started, resources in the form of more social workers were added as part of the social services’ overall promotion of parental support. The managers highlighted these resources as crucial; otherwise, there would not have been enough social workers for each team.

*“I think our managers made the right choice by first expanding the number of staff and then investing in this. So that you don’t just squeeze this in” (Social worker)*

Some participants said that the way of working attracted staff to start working at the family centre. Moreover, they described that the staff already employed there wanted to stay because it was a good way of working, both as a team and because of the feeling that the extended home-visit programme really were benefiting the children and families.

## Discussion

The professionals’ experiences of the extended home-visit programme resulted in the overarching theme *A key to facilitating early support*. The programme and the collaboration it induced, was a key to detecting and supporting families earlier if needed. Therefore, that the extended home-visit programme was offered to all families, not only those living in a socioeconomic vulnerable area [7, 11, 14], was a prerequisite. The extended home-visit programme also aligns with the WHO initiative nurturing care [12]. The driving force was to improve if needed, and if this could help detect any issues or problems earlier, it was appreciated by CHS nurses, social workers, and managers. The collaboration was enhanced by the different professional competencies complementing each other, and by being located at the family centre together. Establishing collaboration and teamwork can be complex [18, 23], so a theoretical framework can be used as support and as a facilitating resource. The Sunnybrook Interprofessional Collaboration Framework [17, 24] is therefore used as a frame in this discussion, since it aims to support interprofessional collaboration, clinical practice, education, improvements, and leadership approaches, which all are important aspects in the micro system foundation [16].

### The core competences in team collaboration

Interprofessional teamwork depends on transparency, openness, and willingness to collaborate [17]. The willingness to work in teams was expressed as a prerequisite for introducing the extended home visit programme. Staff satisfaction and team culture are essential to develop functional teamwork [21]. However, to gain value, the team members must be willing to cooperate and sometimes sacrifice their autonomy [18]. The participants in our study described that the advantages gained and the improved ability to support the families were more significant than the concerns of sharing the time and subject of the visit with another professional. This new way of working strengthened the opportunities to work with the three-tier national CHS programme [6].

Another factor that facilitated the teamwork was that the CHS nurses and social workers were all located at the family centre. Flores and Sarkadi [34] show the difficulties when the staff belong to different organisations and are located at different places. This indicate that the location in the family centre facilitate a collaborative work between CHS nurses and social workers. Research also shows that the integration of health and social services is a strategy widely used internationally to accomplish more sustainable care [35–37].

Both CHS nurses and social workers expressed that openness and the opportunity to participate in the development of the extended home-visit programme were highly appreciated. Leadership and support were vital, which is shown in other studies [34, 35]. Team meetings also allow learning and reflection, according to the review by Körner et al. [21]. Prior to the extended home-visit programme, there was a fear that bringing along a social worker could scare the parents, but the participants did not experience that. The risk of stigmatization when offering an intervention only to a particular group, that is, in a socioeconomically vulnerable area, are highlighted in the research [11, 30]. The decision to offer the extended home-visit programme to all new parents in the municipality was a way to not stigmatize specific families, which seemed to be a useful way to support all families in need of support. Managers in a study by Franzén and Nilsson [38] expressed positive attitudes towards more universal programmes as more families can benefit from home-visits.

Before this extended home-visit programme was initiated, the CHS nurses conducted home-visits on their own, in line with the national CHS programme [4]. The participants expressed that making visits together made contacting the social worker easier for the parents if there were any needs. They could even make an appointment directly during the home-visit. Building relations with the families is essential when working in a voluntary function [30] and it is also a way of establishing a caring relationship, which facilitates providing early support in line with the three-tier national CHS programme [6].

Communication across professions and roles is essential for creating joint processes and a shared understanding; teams must jointly decide on appropriate actions [14, 34]. The possibility of actively developing the extended home-visit programme was highlighted as an advantage for the CHS nurses and the social workers participating in this new way of working. In addition, the managers shed light on the positive experiences of developing the extended home-visit programme step by step together and with support from a project leader, also highlighted in Flores and Sarkadi [34]. The need of supporting structures could on an overall level be seen in the decision about resources and, in more detail, in the schedules and equipment needed to conduct the extended home-visits. The need of supporting structures, time, and sufficient staff resources when working with extended home-visit programmes is also highlighted by other researchers [11, 30, 38]. The fact that the new way of working was introduced in collaboration was highlighted by all participants; the staff and managers planned and co-created the work together. This way of working, building teams prior to introducing a new way of working, can improve collaborative work [18]. The collaboration between the social workers and the CHS nurses in other parts of the family centre’s work was also improved through the work with the extended home-visit programme.

Team collaboration can entail conflict. Team members need skills to create solutions [17], and several examples of a solution-focus are evident in our results. When an extended home-visit programme first was discussed at the family centre, there were concerns about how to become a team. The CHS nurses were used to having home-visits as their assignment, and now they would have to share the home-visit with “their family” with another professional and have someone else watching them. From the social workers’ perspective, it is a question of being part of the team, not just someone coming with the nurse to the family. However, a positive attitude towards this new way of working made it easier to find solutions to the initial difficulties. This positive attitude made them manage challenges on an overall level, and practical questions such as logistics could be solved through reflection and by creating a micro team. Not even the COVID-19 pandemic stopped the progress of the extended home-visit programme, which was the case in other studies, for example the one by Mangrio and Hjortsjö [11]. One supporting structure during the COVID-19 pandemic in the present case was the collaboration with the Department for Communicable Disease Control in the region, helping the staff continue making home-visits in a safe way.

Successful collaboration requires possibilities to reflect and learn from what works and what needs to improve [17]. The participants emphasized that reflection was important. The opportunity to reflect together in the car after the home-visit, sharing your thoughts with someone else, were perceived to add value for the child and family. One benefit of teamwork is not being alone in your decisions, as well as the feeling of having better opportunities to solve problems [18]. Strengthening the family has shown to be essential when preventing health issues in childhood [2], in line with the global intentions expressed by WHO [1, 12], UNICEF [3], and the Swedish National Board of Health and Welfare [4].

Not all micro teams continued the reflection meetings, but initially they were adding value to the collaboration work. Reflecting on the process of working together was shown to be significant [22]. Studies show that collaboration and teamwork across organizational boundaries can be challenging [19, 23] and need support from the management [18]. In this case, the managers were positive, and the work was also supported by a project leader, which all participants thought was one success factor. Florens and Sarkadi [34] describe that having a support person will increase the opportunities to create a sustainable work.

To collaborate, the team members must understand each other’s roles and competencies [17]. In this study, there was no problem with the different professions or roles; on the contrary, coming from different backgrounds and organizations enriched the collaboration and added value for the families. Nygren et al. [26] also show that the team’s expertise exceeded the individual team members, and thereby added value for both professionals and families. Teamwork also facilitated the use of the different roles; the families immediately got a quick response from the “right” person/profession. This was also found in the study by Mangrio and Hjortsjö [11], showing that the collaboration created a deeper understanding of each other’s roles and competences. The different perspectives also developed the work and were useful, helpful, and valuable in supporting the family [37, 39]. The participants helped each other at the visits but could also support each other afterwards and discuss their impressions or any doubts they may have [40].

### The four domains

The framework for interprofessional collaborative teamwork [17] includes 1) interprofessional practice and care, 2) interprofessional leadership, 3) interprofessional research and quality improvement, and 4) interprofessional education. Those domains correspond to our results, as an important issue was the preparation phase, which consisted of joint education in the extended home-visit programme method, essential for well-functioning collaboration [18]. The participants also learned from each other, and their different roles and knowledge added value for themselves and the families. Learning and collaboration are interconnected [23]. Like this study, evaluation is one way of learning from practice in a broader sense. Too often, new ways of working are introduced without or with very little evidence [18]. Therefore, research during the adoption of the extended home-visit programme in Sweden is essential [10, 11, 28]. The results of this study can be useful when introducing extended home-visit programmes further in Swedish CHSs.

The connection to all participants’ willingness to improve for the sake of the child and family is expressed in the overall theme *A key to facilitating early support*, which aligns with interprofessional practice and care. It also illuminates the openness from all participants. Change behaviour can be challenging to achieve [23], but improvement methods can be useful to foster change behaviour, which facilitates collaboration. Leadership was highlighted, as the managers’ ongoing support was essential, which is corresponding to the findings in Franzén and Nilsson [28]. However, the organization also dared to invest resources to allow employees to develop the extended home visit programme in the best possible way. Another study about introducing a home-visit programme conclude that extra resources and long-term planning is essential to succeed [34]. Although, there can be struggle for municipalities to offer an extended home-visit programme to all families. Therefore, this study needs to be followed by examine the effects when offering an extended home-visit programme to all new parents.

### Limitations

This study describes experiences of the introduction of an extended home-visit programme offered to all new parents in the context of a family centre in a municipality. Since the programme was introduced step by step, only one family centre was included, which can be seen as a limitation. In total the participants were few, but all possible staff members, including managers, took part; only one CHS nurse was not part of the focus groups due to sick leave at the day the focus group interview took place. The small sample needs to be taken into consideration when it comes to the transferability of the results. Although, the amount of data was considered rich, and different perspectives arise in the material.

Subjectivity is always an issue in qualitative research and needs to be considered [33]. In this study, one of the researchers was employed by the organization, but the other researcher, who conducted all focus groups interviews, had no connection to the family centre. Both researchers have experience of qualitative analysis. In a reflexive thematic analysis, researchers take an active role in the interpretation of data and thereby, construction of knowledge. At the same time, they need to be aware of their assumptions and positionings to enable credibility and trustworthiness [31, 33].

Focus group interviews are a well-established method when studying working conditions [32], although there are some challenges. The participants can feel uncomfortable discussing their experiences with colleagues present. To minimize risk of discomfort, the focus groups were made up of one staff category at a time. In the interviews, the challenges were explained, and the participants had rich discussions. Quotations, used in the result, is also a way to strengthen the trustworthiness.

## Conclusions

Overall, the extended home-visits were appreciated by all participants, and they were experienced as useful and as adding value for the children and families. To offer extended home-visits to all new parents, not only in socioeconomically vulnerable areas, increase the possibility for early detection of issues, and thereby offer early support.

The precondition of an already existing family centre organization was one of the facilitators, functioning as a meeting point to expand the collaboration. At the same time, flexibility and logistical demands are important to consider making collaboration work, which all the participants emphasized.

## Supplementary Information

Supplementary Material 1.

## Data Availability

All data generated and analysed during this study are included in this published article.

## Abbreviations

CHS: Child Health Services
CHS nurse: Child Health Services nurse
