# Supplementary material for: Collaborative extended home-visits as a key to facilitating early support within the frame of a family centre in Sweden
Source: BMC Health Serv Res. 2024 Dec 3;24:1532. doi: 10.1186/s12913-024-12039-z (PMC11616361; doi:10.1186/s12913-024-12039-z)
Supplement: Supplementary file 1 — Supplementary Material 1. [file 12913_2024_12039_MOESM1_ESM.docx]

**Staff's experiences of extended home visits – focus group interview guide**

**Introduction**

The project with extended home visits, where the families are offered home visits by a child health nurse and social worker together, has been ongoing for a while.

An important part when introducing new ways of working is to evaluate them. This focus group interview is one part of the evaluations, to investigate the staff's and managers thoughts on this way of working.

**Background:**

Education/profession

Experiences of work at family center

Experiences of working with home visits

---------------------------------------------------------------------------------------------

**To discuss:**

- What do you think about families being offered home visits by a CHS nurse and social worker together?
- What is it like to carry out home visits together?
- What is the difference between a home visit and a CHS / family center visit?
- What is the difference between making home visits alone and together with a social worker/CHS nurse?
- Do you think that the CHS nurses, and the social workers different skills interact during the home visit? – If so, how?
- What could improve the design and work with home visits?
- What do you think is important to include in the continued development of extended home visits?
- Is there anything else you would like to share?
